# Integrated Behavioral, Genetic and Brain Circuit Visualization Methods to Unravel Functional Anatomy of Zebrafish Amygdala

**DOI:** 10.3389/fnana.2022.837527

**Published:** 2022-05-25

**Authors:** Pradeep Lal, Koichi Kawakami

**Affiliations:** ^1^Integrative Fish Biology Group, Climate and Environment Department, NORCE Norwegian Research Centre, Bergen, Norway; ^2^Division of Molecular and Developmental Biology, National Institute of Genetics, and Department of Genetics, Graduate University for Advanced Studies (SOKENDAI), Mishima, Japan

**Keywords:** zebrafish, amygdala, Gal4-UAS system, emotion, dorsomedial telencephalon, pallium, subpallium, transgenic

## Abstract

The mammalian amygdala is a complex forebrain structure consisting of a heterogeneous group of nuclei derived from the pallial and subpallial telencephalon. It plays a critical role in a broad range of behaviors such as emotion, cognition, and social behavior; within the amygdala each nucleus has a distinct role in these behavioral processes. Topological, hodological, molecular, and functional studies suggest the presence of an amygdala-like structure in the zebrafish brain. It has been suggested that the pallial amygdala homolog corresponds to the medial zone of the dorsal telencephalon (Dm) and the subpallial amygdala homolog corresponds to the nuclei in the ventral telencephalon located close to and topographically basal to Dm. However, these brain regions are broad and understanding the functional anatomy of the zebrafish amygdala requires investigating the role of specific populations of neurons in brain function and behavior. In zebrafish, the highly efficient *Tol2* transposon-mediated transgenesis method together with the targeted gene expression by the Gal4-UAS system has been a powerful tool in labeling, visualizing, and manipulating the function of specific cell types in the brain. The transgenic resource combined with neuronal activity imaging, optogenetics, pharmacology, and quantitative behavioral analyses enables functional analyses of neuronal circuits. Here, we review earlier studies focused on teleost amygdala anatomy and function and discuss how the transgenic resource and tools can help unravel the functional anatomy of the zebrafish amygdala.

## Introduction

The amygdala is a closely associated cluster of nuclei found in the cerebral hemispheres of vertebrates; it is located within the temporal lobe of primates and in the caudoventral telencephalon of non-primate mammals (Goddard, 1964; Maren, 2001). It is best known for its role in recognition and response to fearful stimuli. Initial evidence of its involvement in emotional response to stimuli comes from lesion studies in non-human primates; temporal lobe lesion in monkeys (*Macaca mulatta*) resulted in a marked change in emotion including loss of fear (Brown and Schäer, 1888; Kluver and Bucy, 1997). Subsequently, Weiskrantz (1956) showed that this loss of fear results from damage to the amygdala region. Studies in several mammalian species such as rodents, cats, rabbits, dogs, and humans showed functional similarity of the amygdala across mammals (Goddard, 1964; Blanchard and Blanchard, 1972; Adolphs et al., 1994; Anderson and Phelps, 2001). Although morphological differences exist, the circuits and function of the amygdala are well conserved; amygdalar lesion results in an inability to recognize and respond to fear stimuli (LeDoux, 2000; Maren, 2001).

The amygdala is derived from both pallial and subpallial cells (Puelles et al., 2000). It consists of approximately 20 subnuclei, divided into three major groups; namely, cortical nuclei, basolateral nuclei (BLA), centromedial nuclei (medial [MeA], and central [CeA], and bed nucleus of the stria terminalis [BNST]) (Sah et al., 2003; Marek et al., 2013). Each subnucleus is characterized by distinct neural types and connections; the robustness of fear response has allowed understanding the role of the amygdala subnuclei in specific behavioral responses. The BLA is a cortex-like structure containing predominantly glutamatergic neurons and is characterized by interconnections with the hippocampus and the cortical areas; it is the main sensory interface of the amygdala. The CeA is a striatum-like structure containing predominantly GABAergic neurons and is the primary output structure that has divergent projections to BNST and other brain regions; it controls emotional responses (LeDoux, 2000; Maren, 2001; Marek et al., 2013). The cortical and medial nuclei are characterized by major projections from the main and accessory olfactory bulbs (Sah et al., 2003). In-depth reviews of structure and function of the amygdala can be found elsewhere (Sah et al., 2003; Ehrlich et al., 2009; Duvarci and Pare, 2014; Janak and Tye, 2015; Tovote et al., 2015).

The mammalian amygdala is crucial for a broad range of emotional and motivational behaviors such as fear learning and responses, social behavior, anxiety-related behavior, and addiction (LeDoux, 2000; Tovote et al., 2015). Studies show existence of amygdala-like brain regions in non-mammalian vertebrates including teleost fish (Martínez-García et al., 2009). For a long time, amygdala-like structure in teleost had remained elusive. A series of lesion studies together with behavioral experiments in goldfish showed that distinct brain regions in teleost brain mediate specific behavioral responses and identified forebrain regions that mediate fear conditioning (Rodríguez et al., 2002a,b; Salas et al., 2003; Portavella et al., 2004a,b; Broglio et al., 2005). Further developmental, hodological, and functional studies in teleosts including zebrafish revealed the presence of an amygdala-like structure consisting of the medial zone of the dorsal telencephalon (Dm) and the dorsal most nucleus (Vdd), supracommissural nucleus (Vs), postcommissural nucleus (Vp), and intermediate nucleus (Vi) of the ventral telencephalon (Northcutt, 2006; Lau et al., 2011; Ganz et al., 2014; von Trotha et al., 2014; Biechl et al., 2017; Porter and Mueller, 2020). This review focuses on the past studies on the presence of an amygdala-like structure in zebrafish and the recent advances and future perspectives on understanding the functional anatomy of zebrafish amygdala.

## The Zebrafish Amygdala Complex

**Figure 1:**
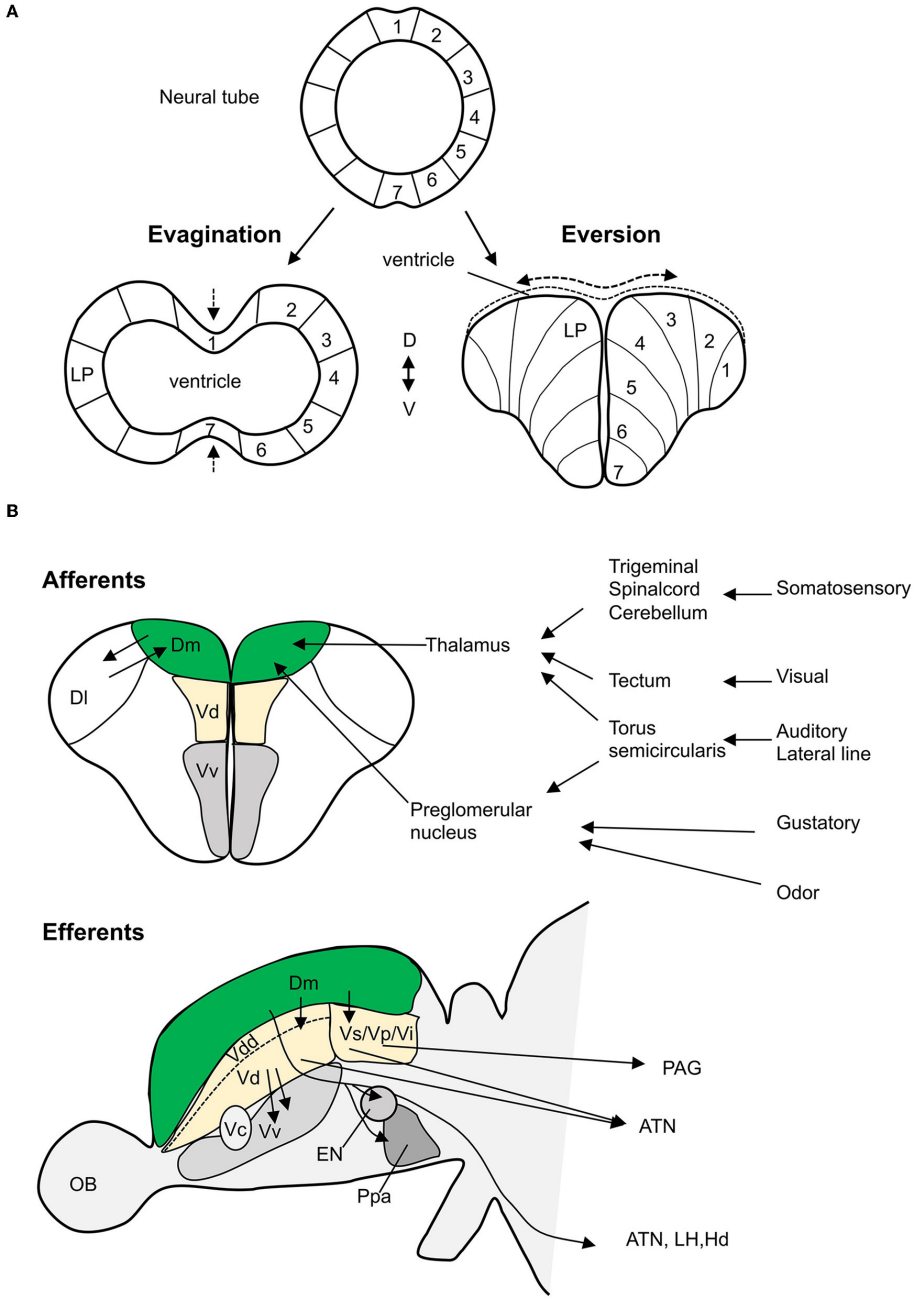
Teleost amygdala. **(A)** The development of telencephalon from neural tube (top) comparing evagination (in mammals) *vs*. eversion (in teleosts). In mammals, the lateral pallium (LP), putative amygdala region, lies laterally, whereas in teleosts, in a simple eversion, the corresponding structure lies medially. **(B)** The main neural connectivity of the teleost amygdala complex. Teleost Dm receives sensory information (somatosensory, visual, auditory, lateral line, gustatory, and odor) through thalamus and preglomerular complex (coronal view/top panel). Dm has extensive projections (sagittal view/bottom panel) to the dorsal nucleus (Vd), suppracommissural nucelus (Vs), and postcommissural nucelus (Vp) of the ventral telencephalon. These nuclei project efferents to several hypothalamic nuclei and the periaqueductal gray (PAG). Olfactory projections to Vd, Vs, and Vi are not showed. The dorsal most nucleus (Vdd) is dorsal most part of the ventral telencephalon as defined by Porter and Mueller (2020). ATN, anterior tuberal nucleus; Dl, dorsolateral telencephalon; EN, entopeduncular nucleus; Hd, dorsal zone of periventricular hypothalamus; LH, lateral hypothalamus; OB, olfactory bulb; Ppa, preoptic area; Vi, the intermediate nucleus of the ventral telencephalon (adapted from Nieuwenhuys and Meek, 1990; Folgueira et al., 2004; Lal et al., 2018, and Northcutt, 2006).

Similar to mammalian telencephalon, teleost telencephalon is divided into a dorsal telencephalon or pallium and a ventral telencephalon or subpallium (Wullimann and Mueller, 2004). However, due to differences in developmental mechanisms, there are marked differences in the organization of pallial nuclei between mammals and teleosts. In mammals, the forebrain is formed by evagination, whereas the teleost pallium is formed by eversion (Nieuwenhuys and Meek, 1990; Braford, 1995; Nieuwenhuys, 2009) (Figure 1A). Eversion leads to an inverted medio-lateral organization of pallium in teleost compared to mammals; hence, the medial and lateral regions in teleost forebrain are suggested to correspond to the lateral and medial regions in mammalian forebrain, respectively. Studies in zebrafish have visualized the eversion and show that eversion process is more complex than a simple out-folding of the neural tube (Folgueira et al., 2012); pallial growth, neuronal differentiation, and radial migration of neurons add complexity to the pallial organization (Mueller et al., 2011; Folgueira et al., 2012; Dirian et al., 2014; Furlan et al., 2017). This presents difficulty in establishing homologies between brain regions in mammals and zebrafish.

The expression pattern of conserved developmental regulatory genes and molecular markers provides useful landmarks to identify subdivisions within a brain region and to homologize the brain regions among divergent vertebrate species. In mammals, the developing pallium expresses *Tbr1, Lhx9*, and *Emx1* genes; the pallial amygdala is marked by the expression of *Tbr1* but the absence of *Emx1* gene (Rétaux et al., 1999; Puelles et al., 2000). Interestingly, the excitatory neurons of the BLA are produced from the pallial *Emx1*-expressing cell lineage (Gorski et al., 2002). The developing zebrafish pallium expresses *tbr1, lhx9*, and *emx-*genes (Mione et al., 2001; Costagli et al., 2002; Ganz et al., 2014; Liu et al., 2015) and there is no distinct *emx*-negative pallial region (Ganz et al., 2014). In agreement with the pallial location, most neurons in the Dm are glutamatergic (Wullimann and Mueller, 2004; von Trotha et al., 2014). Furthermore, zebrafish Dm expresses cannabinoid receptor (*cb1*) (Lam et al., 2006), a marker for BLA in rodents (Mailleux and Vanderhaeghen, 1992; Matsuda et al., 1993; Katona et al., 2001). Taken together, the zebrafish Dm is suggested to be the homolog of mammalian BLA.

The subpallial homologs, MeA, CeA, and BNST, are predicted to be close to and topographically basal to the Dm in the subpallium consisting of Vdd, Vs, Vp, and Vi of the ventral telencephalon (Mueller et al., 2008; Ganz et al., 2012; Biechl et al., 2017; Porter and Mueller, 2020). The mammalian subpallial amygdala is characterized by the expression of *Dlx2* and *GAD67* as well as *Nkx2.1, Lhx6, Lhx7*; in addition, amygdalar cells those originating from the striatal area express *Isl1* (Puelles et al., 2000; Moreno et al., 2009, 2018). The zebrafish-developing dorsal subpallium expresses *dlx2, lhx6, lhx7*, and *gad1b* (Mueller et al., 2008). In adult zebrafish, the subpallium broadly expresses *dlx2a, dlx5a*, and *gad1b*; the expression of *lhx6* and *lhx7* are restricted to Vs; the dorsal Vs and most of Vp lack *nkx2.1b* and *lhx1b* expression; medial and ventral parts of Vs express *isl* (Ganz et al., 2012). The Vdd also lacks expression of *isl1* (Baeuml et al., 2019; Porter and Mueller, 2020). Based on this, Vdd, dorsal and medial Vs, and most of Vp have been proposed to constitute the central amygdala (Ganz et al., 2012; Porter and Mueller, 2020). In mice, among the subpallial amygdalar nuclei, the BNST expresses *Nkx2.1* (Puelles et al., 2000; García-López et al., 2008). In zebrafish, the ventral-most part of Vs and Vp expresses *nkx2.1b* suggesting homology to the mammalian BNST (Ganz et al., 2012). In mammals, *otp* is a differential marker of MeA within the telecephalon; based on neuroanatomical features and expression pattern of the *otpa* gene, the *otpa*-positive neuronal population in Vi is identified as the homolog of MeA (Biechl et al., 2017). Calretinin is another differential marker of MeA (Wójcik et al., 2013), Porter and Mueller (2020) identified the calretinin-expressing regions within the Vs, Vp, Vdd, and Vi as the MeA homolog. Although the identified region includes the *otpa-*expressing cells in the Vi, this study interpreted Vi to be a thalamic eminence (EmT) derivative as it is contiguous to EmT and predominantly glutamatergic. In mammals, the glutamatergic cells in the MeA originate from the ventral pallium and the hypothalamic supraopto-paraventricular area (Morales et al., 2021); a similar origin for excitatory cells in the zebrafish Vi has been suggested that Vi is a part of the MeA homolog (Gerlach and Wullimann, 2021).

## Conserved Neural Connectivity Between Zebrafish and Mammalian Amygdala

Although the developmental and molecular studies delineated subdivisions within the zebrafish amygdala, it does not imply that the identified subdivisions have similar functional relationships with other brain structures (Faunes et al., 2015). The hodological studies provide compelling evidence on similarity in neuronal architecture and homology between the teleost and the mammalian amygdala (Figure 1B). In mammals, BLA receives primary sensory inputs such as visual, auditory, olfactory, gustatory, and somatosensory from the sensory thalamus and cortex (Maren, 2001; Ledoux, 2003). Tracing studies in goldfish show that Dm receives afferent projections from the preglomerular complex of the posterior tuberculum and the central nucleus of the thalamus (Northcutt, 2006). The preglomerular complex receives auditory input *via* the medial and central pretoral nuclei, lateral line input *via* the ventrolateral toral nucleus, visual input *via* the optic tectum, and chemosensory information *via* the medial preglomerular nucleus (Northcutt, 2006). In goldfish, the central posterior nucleus of the thalamus receives and responds to auditory and visual inputs (Kirsch et al., 2002; Northcutt, 2006). In the zebrafish larvae, the dorsal pallium including the putative Dm region is activated in response to light pulses (Randlett et al., 2015), and the thalamus responds to auditory stimuli (Constantin et al., 2020). In goldfish (Kato et al., 2012) and trout (Folgueira et al., 2004), Dm receives gustatory cues from the preglomerular tertiary gustatory nucleus. However, a study on zebrafish could not find similar connectivity (Yáñez et al., 2017). In juvenile and adult zebrafish, calcium imaging studies show that Dm is activated in response to odors (Diaz-Verdugo et al., 2019; Bartoszek et al., 2021). Thus, the teleost Dm receives sensory input similar to the mammalian BLA.

In mammals, BLA and hippocampus are connected by bidirectional projections (Maren, 2001). Similarly, tracing studies in goldfish and trout show that the homolog of the hippocampus in fish, the lateral zone of the dorsal telencephalon (Dl), forms projections to the Dm (Folgueira et al., 2004; Northcutt, 2006).

In mammals, the intra-amygdalar projections convey information from BLA to CeA. The CeA has divergent connections to hypothalamus and brainstem that are known to modulate the physiological responses during fear conditioning (Royer et al., 1999; Maren, 2001). Anatomical studies in goldfish, trout, and zebrafish show that the Dm has efferent projections to the subpallial homologs, Vd, Vs, and Vp (Folgueira et al., 2004; Northcutt, 2006; Lal et al., 2018). In trout, tracing studies show that Vd is well connected to Vs, and both Vd and Vs have efferent projections to several hypothalamic nuclei including the anterior tuberal nucleus (Folgueira et al., 2004). In midshipman fish, connectivity between Vs and the periaqueductal gray homolog has been shown (Kittelberger and Bass, 2013). In mammals, CeA is the main output nucleus and regulates fear response. In teleosts, Dm has major efferent projections to several brain regions such as the lateral hypothalamus, the anterior tuberal nucleus, and the dorsal zone of the periventricular hypothalamus, that are proposed to modulate fear response, presenting a teleost specific difference (Northcutt, 2006; Lal et al., 2018; Yáñez et al., 2021).

The mammalian MeA receives projections from the vomeronasal organ and is involved in social and fear odor detection (O'Connell and Hofmann, 2011). Although teleosts lack a vomeronasal organ (Døving and Trotier, 1998), crypt cells in the zebrafish olfactory epithelium have shown increased pERK level in response to kin odor (Biechl et al., 2016). Crypt/microvillous sensory neurons project to the mediodorsal olfactory bulb glomerulus which has efferent projections to the ventral telencephalon including Vi. Vi shows increased pERK level in response to kin recognition and projects to the tuberal nucleus in the hypothalamus supporting the homology of Vi to the mammalian MeA (Biechl et al., 2017; Gerlach and Wullimann, 2021).

## Functional and Behavioral Role of the Zebrafish Amygdala

Functional and behavioral studies in teleost have mostly focused on the pallial homolog Dm. Portavella et al. (2004a) carried out lesion studies on Dm in goldfish; goldfish were trained in active avoidance fear conditioning and after conditioning Dm was surgically lesioned. The Dm lesioned goldfish showed a deficit in retention of active avoidance response (Portavella et al., 2004a). In a subsequent lesion study, the Dm was showed to be essential for the avoidance learning (Portavella et al., 2004b). A similar ablation study showed that Dm lesion also impairs the acquisition of conditioned taste avoidance (Martín et al., 2011).

In zebrafish, the function of Dm has been analyzed in light/dark choice, drug seeking behavior, innate, and conditioned fear response assays. Light/dark choice task is thought to induce anxiety-like responses in zebrafish (Maximino et al., 2010). Lau et al. (2011) found an increased c-fos expression in Dm and other brain regions, including hypothalamus and ventral telencephalic regions when fish performed a light/dark choice task. von Trotha et al. (2014) analyzed the c-fos expression in the zebrafish brain induced by acute administration of amphetamine and observed increase in c-fos expression in the Dm area during amphetamine-induced place preference behavior. Calcium imaging studies showed that the Dm is activated during aversive reinforcement learning (Aoki et al., 2013). Both rewarding (food) and fear inducing (skin extract) odor activate neurons in the Dm (Ruhl et al., 2015; Diaz-Verdugo et al., 2019; Bartoszek et al., 2021). Recently, using genetic methods, we identified a population of neurons, named 120A-Dm-neurons, mediating fear conditioning; these neurons were also found to modulate freezing in response to skin extract, an innate fear cue (Lal et al., 2018). Thus, like mammalian amygdala, zebrafish Dm is involved in emotional and motivational behaviors.

## Investigating the Functional Anatomy of the Zebrafish Amygdala

**Figure 2:**
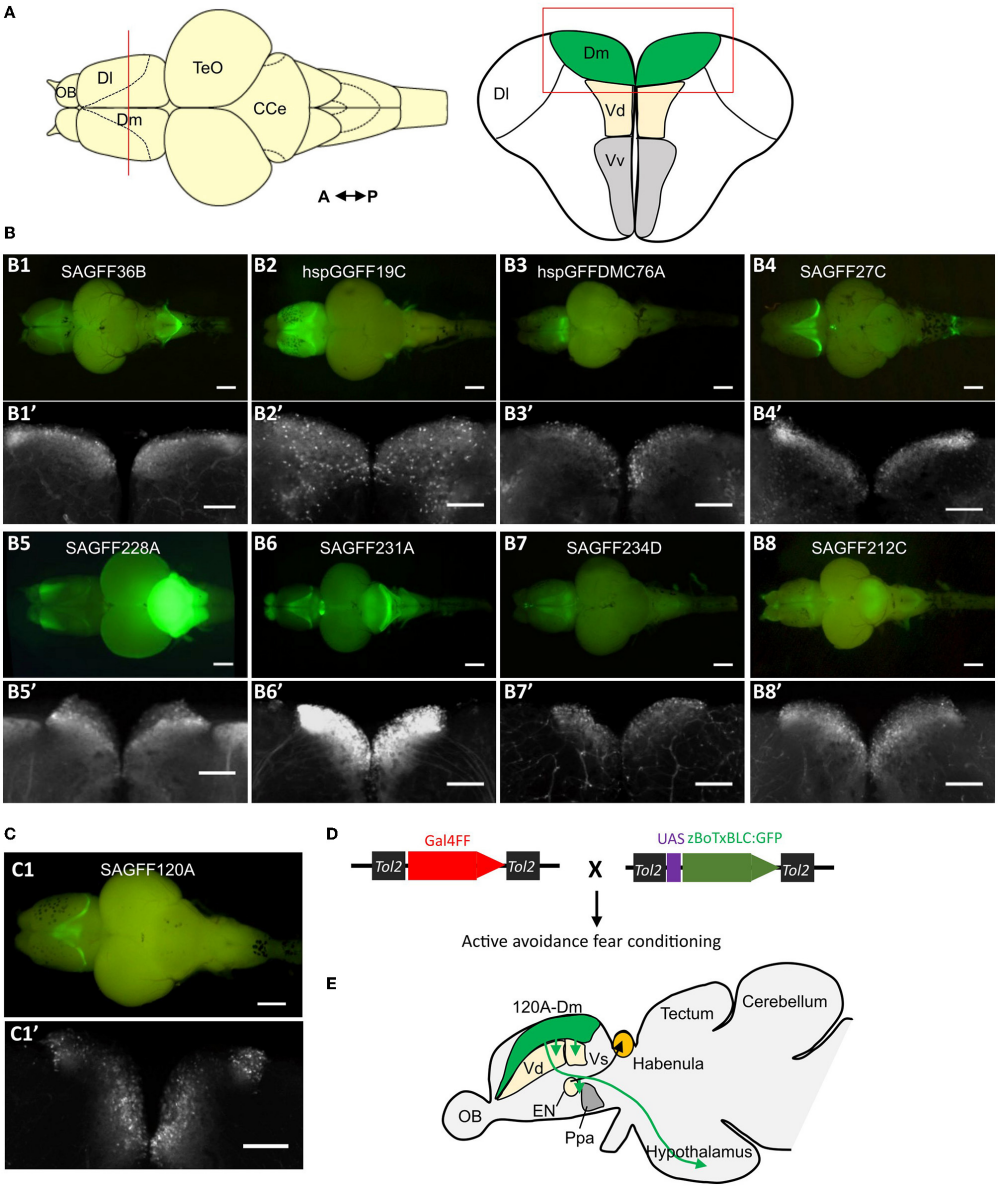
Genetic dissection of the zebrafish amygdala. **(A)** Schematic of dorsal and coronal view of the zebrafish brain and telencephalon, respectively. The red line in the dorsal view shows the position of coronal section. **(B)** Examples of isolated brains of gene trap and enhancer trap transgenic zebrafish lines that show Gal4FF (UAS:GFP) expression in the Dm region. **(B1–B8)** Dorsal view of the isolated brain. Scale bar, 500 μm. **(B1'–B8')** Coronal section of the corresponding transgenic fish in **(B1–B8)**. The position and region of the coronal section are showed in A (red line and red box). Scale bar, 200 μm. **(C)** The Gal4FF (UAS:GFP) expression pattern in the brain of transgenic fish SAGFF120A. SAGFF120A is an *emx3*-enhancer trap line and labels a population of neurons in the Dm, named 120A-Dm-neurons. **(C1)** Dorsal view. Scale bar, 500 μm. **(C1')** Coronal view as above. Scale bar, 200 μm. **(D)** Gal4FF drivers lines are crossed with botulinum toxin effector fish (UAS:zBoTxBLC:GFP). The double transgenic fish is analyzed in an active avoidance fear conditioning assay. **(E)** 120A-Dm-neurons have efferent projections to Vd, Vs, EN, Ppa, and hypothalamus. CCe, corpus cerebelli; Dl, dorsolateral telencephalon; EN, entopeduncular nucleus; OB, olfactory bulb; Ppa, preoptic area; TeO, optic tectum; Vd, the dorsal zone of the ventral telencephalon; Vv, the ventral zone of the ventral telencephalon [figures reused and adapted from Lal et al. (2018), https://creativecommons.org/licenses/by/4.0/].

The zebrafish amygdala is a broad region and constitutes genetically diverse populations of neurons; evolutionary molecular studies have revealed distinct subdivisions within this structure (Ganz et al., 2012, 2014; Porter and Mueller, 2020). Although the functional and ablation studies provide evidence of homology, to understand the functional organization of the zebrafish amygdala, it is crucial to study the structure and function of its distinct populations of neurons in a particular behavioral task. Genetic methods allow target specificity in labeling, visualizing, and manipulating the activity of specific neurons (Scott et al., 2007; Asakawa et al., 2008). In a recent study, by using genetic methods, we identified and mapped neural circuits-mediating fear conditioning (Lal et al., 2018). Using the *Tol2* transposon-mediated gene and enhancer trap methods and the Gal4-UAS binary gene expression system, we performed a large-scale genetic screen and isolated transgenic fish lines that expressed Gal4FF, an engineered Gal4 transcription activator, in specific populations of cells in the brain including the amygdala region (Figures 2A–C). These transgenic lines were crossed with an UAS effector fish carrying genetically engineered botulinum neurotoxin (UAS:zBoTxBLC:GFP) (Figure 2D). In these fish, the botulinum neurotoxin is expressed in the Gal4FF expressing cells and inhibits the activity of the labeled neurons (Sternberg et al., 2016). These double transgenic fish were analyzed in an active avoidance fear conditioning assay. We identified several fish lines that had Gal4FF UAS:zBoTxBLC:GFP expression in the Dm and showed a defect in acquisition of active avoidance fear conditioning. In two fish lines, the Gal4FF expression was restricted to a population of neurons within the Dm area, named 120A-Dm-neurons (Figure 2C). Inhibition of 120A-Dm-neurons also reduced performance in Pavlovian fear conditioning suggesting that these neurons mediate sensory association for conditioned fear. Moreover, these neurons were also found to modulate freezing in response to skin extract, an innate fear response. Thus, the 120A-Dm-neurons modulate both conditioned and innate fear response.

The transgenic approach allowed neurochemical and anatomical characterization of the labeled neurons. The 120A-Dm-neurons expressed *vglut* genes and were glutamatergic. The 120A-Dm-neurons had efferent projections to various brain structures including the subpallial amygdala homologs Vd and Vs, entopeduncular nucleus (EN), preoptic region, and the hypothalamus (Figure 2E). The Dm-hypothalamus pathway is suggested to modulate fear response. The zebrafish EN consists of a dorsal GABAergic part (ENd) and a ventral glutamatergic nucleus (ENv). The teleost ENd and ENv are proposed to be the homologs of the EN of non-primate mammals and the bed nucleus of stria medullaris, respectively (Mueller and Guo, 2009). In goldfish and zebrafish, ENv has efferent projections to habenula. The habenula-median raphe circuit in zebrafish is essential for active avoidance conditioning (Amo et al., 2014). Hence, the Dm–EN circuit may mediate active avoidance response. Thus, the genetic approach helped identify a functional neuronal circuit in the Dm essential for fear conditioning.

## Perspectives

In summary, studies have identified the amygdala homolog in zebrafish. However, to understand its functional anatomy, it is crucial to understand how it contributes to the generation of different behaviors. To understand the structure and function of distinct populations of neurons, genetic approaches provide target specificity in labeling, visualization, and manipulation of specific neural circuits (Scott et al., 2007; Asakawa et al., 2008; Förster et al., 2017; Lal et al., 2018). However, it is unlikely that all the neurons that share a genetic marker or that have common projection patterns also have identical functions. There is a need for an even greater library of molecular markers and transgenic resources that can be utilized to achieve target specificity, e.g., by using combinatorial expression systems such as Gal4-UAS and Cre/loxP (Sato et al., 2007).

Moreover, how information is processed within the zebrafish amygdala and how this affects the downstream neural circuit is not known. To address this, we need to simultaneously manipulate the specific neural populations within the amygdala and observe the activity of the target brain regions. Recently developed virtual reality systems allow *in vivo* imaging of adult zebrafish brain at a single-cell resolution during a behavioral task (Huang et al., 2020; Torigoe et al., 2021). The optogenetic and chemogenetic approaches combined with neural activity imaging are needed to study the role of specific populations of neurons in neural computations (Antinucci et al., 2020). Although the amygdala is well known for processing and response to fear stimuli, it is also crucial for a broad range of behaviors such as emotion, cognition, and social behavior (Murray, 2007; Morrison and Salzman, 2010; O'Connell and Hofmann, 2011; Janak and Tye, 2015). An integrated approach using the transgenic resource and tools combined with neuronal activity imaging, neural activity manipulation, and quantitative behavioral analyses will help to reveal the functional anatomy of the zebrafish amygdala.

## Author Contributions

PL wrote the manuscript and generated the figures with inputs from KK. Both authors approved the submitted version.

## Conflict of Interest

The authors declare that the research was conducted in the absence of any commercial or financial relationships that could be construed as a potential conflict of interest.

## Publisher's Note

All claims expressed in this article are solely those of the authors and do not necessarily represent those of their affiliated organizations, or those of the publisher, the editors and the reviewers. Any product that may be evaluated in this article, or claim that may be made by its manufacturer, is not guaranteed or endorsed by the publisher.
